# The relationship between changes in alcohol consumption and hepatic steatosis among alcohol consumers: a large-scale population-based Biobank study

**DOI:** 10.3389/fnut.2025.1647225

**Published:** 2025-10-16

**Authors:** Suosu Wei, Honglin Luo, Zhemin Liu, Fei Liu, Zhong Tang, Pinghua Zhu, Chunxia Deng, Shenhong Qu, Tengyan Wu

**Affiliations:** ^1^Institute of Oncology, Guangxi Academy of Medical Sciences and the People’s Hospital of Guangxi Zhuang Autonomous Region, Nanning, Guangxi, China; ^2^Department of Health Service Management, School of Information and Management, Guangxi Medical University, Nanning, Guangxi, China; ^3^Department of Scientific Cooperation of Guangxi Academy of Medical Sciences and the People’s Hospital of Guangxi Zhuang Autonomous Region, Nanning, Guangxi, China; ^4^Research Center of Medical Sciences, Guangxi Academy of Medical Sciences and the People’s Hospital of Guangxi Zhuang Autonomous Region, Nanning, Guangxi, China; ^5^Department of Humanities and Social Sciences, Guangxi Medical University, Nanning, Guangxi, China; ^6^Department of Endocrinology and Metabolism, Guangxi Academy of Medical Sciences and the People’s Hospital of Guangxi Zhuang Autonomous Region, Nanning, Guangxi, China

**Keywords:** alcohol consumption, hepatic steatosis, epidemiology study, cross-sectional study, Biobank

## Abstract

**Background:**

The relationship between changes in alcohol consumption and hepatic steatosis among alcohol consumers remains poorly understood. This study aimed to evaluate the association between changes in alcohol consumption and hepatic steatosis in a large population-based cohort of alcohol consumers.

**Methods:**

This study included 33,427 participants with reported alcohol consumption, categorized as mild, moderate, or heavy at baseline and imaging visits. Hepatic steatosis was assessed via magnetic resonance (MR) imaging during the imaging visit.

**Results:**

9,131 (27.3%) participants were diagnosed with hepatic steatosis at imaging visit. After adjusting for confounders, mild drinkers who progressed to moderate (aOR 1.26, 95% CI 1.10–1.44) or heavy drinking (aOR 1.70, 95% CI 1.12–2.57) had elevated odds of hepatic steatosis compared to stable mild drinkers. Moderate drinkers who maintained moderate drinking (aOR 1.36, 95% CI 1.21–1.53) or progressed to heavy drinking (aOR 2.27, 95% CI 1.84–2.79) also showed increased risk compared to those who transitioned to mild drinking. Conversely, heavy drinkers who transitioned to moderate (aOR 0.58, 95% CI 0.47–0.72) or mild drinking (aOR 0.34, 95% CI 0.25–0.45) had significantly lower odds compared to stable heavy drinkers. Stratified analyses revealed that males, individuals under 65 years, those with higher BMI, and hypertensive patients were more susceptible to hepatic steatosis with increased alcohol consumption.

**Conclusion:**

Increasing alcohol intake raises the odds of hepatic steatosis, while reducing intake lowers the odds. Public health strategies should focus on decreasing alcohol consumption to alleviate the burden of hepatic steatosis.

## 1 Introduction

Metabolic dysfunction-associated steatotic liver disease (MASLD), previously known as nonalcoholic fatty liver disease, has emerged as a leading global cause of chronic liver disease and a major driver of cirrhosis worldwide (1–3). Concurrently, the COVID-19 pandemic has exacerbated high-risk alcohol consumption, significantly increasing the prevalence of alcohol-related liver disease (ALD) and alcohol-related cirrhosis, particularly in the United States (4–6). This dual burden of metabolic and alcohol-related liver diseases has created a complex epidemiological landscape, with alcohol-related cirrhosis now surpassing other etiologies as the primary indication for liver transplantation among U.S. adults (7, 8). These trends underscore the urgent need for comprehensive strategies to address the growing burden of both MASLD and ALD in this evolving era of liver disease management.

Among individuals with MASLD, concurrent moderate to heavy alcohol consumption is associated with accelerated liver fibrosis progression and more rapid disease advancement. While excessive alcohol intake is a well-established risk factor for hepatic steatosis (9), the relationship between low-to-moderate alcohol consumption and liver health remains controversial. Some studies suggest a potential protective effect, indicating that low-to-moderate alcohol intake may be associated with reduced risks of hepatic steatosis and secondary liver disease. Notably, studies conducted in Japan have shown that moderate alcohol consumption may protect against the development of fatty liver disease (FLD) (10, 11). This geographical variation is supported by a meta-analysis by Roerecke et al. (12) which identified beneficial effects of low alcohol consumption in Japan based on robust epidemiological evidence, although no such association was found in other regions. Further reinforcing this perspective, Dunn et al. (13) reported that moderate alcohol consumption correlated with a lower prevalence of steatohepatitis, hepatocellular ballooning, and liver fibrosis. Similarly, Unalp-Arida and Ruhl (14) found that moderate alcohol consumption was associated with lower controlled attenuation parameter (CAP) levels, while heavy drinking showed no correlation with fatty liver severity. However, contrasting evidence highlights the potential risks of alcohol consumption. Multiple studies have demonstrated that alcohol intake significantly increases the risk of chronic liver diseases, with daily drinking and non-meal-associated consumption posing particularly high risks. Additionally, moderate alcohol consumption has been linked to progressive liver fibrosis, with diabetic patients displaying heightened susceptibility to advanced fibrosis even at moderate drinking levels (15–19). These conflicting findings underscore the complex relationship between alcohol consumption and liver health in patients with MASLD.

To the best of our knowledge, there are currently no large population-based cohort studies that have evaluated the impact of changes in alcohol consumption status on hepatic steatosis. Given the lack of relevant data and the conflicting findings regarding the effect of concurrent alcohol use on the risk of liver disease progression, our objective was to assess the impact of changes in alcohol consumption status on hepatic steatosis.

## 2 Materials and methods

### 2.1 Study design and participants

**Figure 1:**
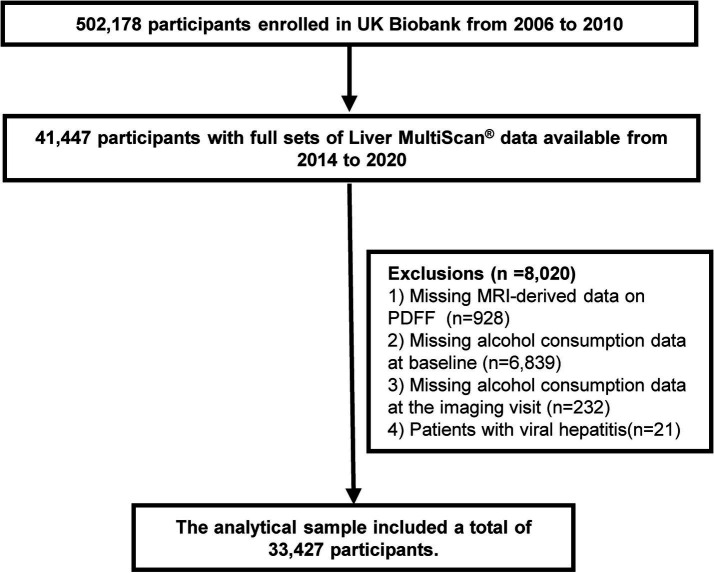
Flowchart of participant selection. MRI, magnetic resonance imaging; PDFF, proton density fat fraction.

This cross-sectional study was performed using the data obtained from the UK Biobank (UKB) dataset (reference number 170239), a large prospective cohort study that recruited over 500,000 participants aged 37–73 years across England, Scotland, and Wales between 2006 and 2010 (20). During the baseline visit, participants underwent an initial assessment, which included information from questionnaires, verbal interviews, physical measurements, and biological samples. In April 2014, a subset of 100,000 participants was invited to participate in the initial phase of a multimodal imaging visit, during which they underwent abdominal magnetic resonance imaging (MRI) (21). Participants who completed the alcohol consumption questionnaire and provided information on their current alcohol intake were included in this study. Of the 502,178 participants, we excluded those who without full sets of Liver MultiScan^®^ data (*n* = 460,731), those with missing data for proton density fat fraction (PDFF) (*n* = 928), those lacking alcohol consumption data at baseline (*n* = 6,839) or alcohol consumption data at the imaging visit (*n* = 232), and those diagnosed with viral hepatitis (*n* = 21). Ultimately, the analytic sample comprised 33,427 participants (Figure 1).

The UK Biobank study received approval from the North West Multicenter Research Ethics Committee (Ref: 11/NW/0382), and all participants provided written informed consent. Information regarding the methods, data availability, and access procedures for the UK Biobank can be found on the study’s website.^1^

### 2.2 Exposure assessment

Alcohol consumption patterns were assessed using a validated questionnaire that systematically evaluated both the frequency and quantity of various alcoholic beverages consumed by participants. Data collection took place at two key time points: the baseline visits (2006–2010) and the subsequent imaging visits (2014). The assessment included standardized measurements for multiple beverage types, such as beer, cider, champagne, white wine, red wine, spirits, and other alcoholic drinks. In this study, participants quantified their average alcohol intake using standardized units, reporting it as weekly consumption for regular drinkers and monthly consumption for occasional drinkers.

Weekly and monthly alcohol consumption was calculated by analyzing the reported frequency and quantity of each beverage type, which were then converted into daily grams of alcohol. The calculations considered the average alcohol by volume (ABV) for each drink along with standard conversion factors to translate beverage units into grams of pure alcohol. The UK Biobank defined alcohol units as follows: a pint or can of beer, lager, or cider is two units; a single shot of spirits (25 mL) is one unit; and a standard glass of wine (175 mL) is two units. Total weekly units were calculated by summing the reported consumption across all categories. For participants reporting monthly intake, the values were converted to weekly units by dividing by 4.3. Finally, weekly units were divided by 7 to determine daily alcohol consumption (22).

Alcohol intake can be broadly classified as mild (up to 20 g per day for women and 30 g for men), moderate (21–39 g per day for women and 31–59 g for men), or heavy (≥40 g per day for women and ≥60 g for men) (23).

### 2.3 Hepatic steatosis definition

Liver MRI scans were conducted following a standardized protocol during the imaging visit. Details of the liver MRI and analysis protocols have been described previously (21, 24). Participants were scanned using a Siemens 1.5 Tesla MAGNETOM Aera scanner (Siemens Healthineers, Erlangen, Germany) with a 6-min dual-echo Dixon Vibe protocol. This procedure produced a volumetric dataset that separated water and fat from the neck to the knee. Body composition analyses were performed using AMRA Profiler Research (AMRA Medical AB, Linköping, Sweden) (25). The average liver PDFF was calculated from nine regions of interest, which were carefully selected to avoid inhomogeneities, major blood vessels, and bile ducts. The presence of steatosis was evaluated using MRI-derived PDFF measurements, which have demonstrated reliability and accuracy in quantifying liver fat content. Hepatic steatosis was defined as a PDFF of ≥5%, consistent with established cut-off values for diagnosing this condition (26, 27).

### 2.4 Covariates

In our analyses, we incorporated demographic data from the UK Biobank dataset, including age, sex, and body mass index (BMI) at both baseline and the imaging visit as potential covariates. BMI ≥30 kg/m^2^ is defined as obesity (28). Furthermore, we conducted a supplementary evaluation of blood biomarkers, including high-density lipoprotein (HDL), low-density lipoprotein (LDL), glycated hemoglobin (HbA1c), triglycerides, and albumin. Additionally, we performed a comprehensive analysis of previously identified risk factors, such as the use of medications for hypertension, diabetes, and dyslipidemia (29). We included metabolic syndrome (MetS) as an important covariate in our analysis. MetS is defined as if participants had three or more of the following criteria (30): (1) abdominal obesity: WC >102 cm for males and >88 cm for females; (2) elevated blood pressure: systolic blood pressure (SBP) ≥130 mmHg or diastolic blood pressure (DBP) ≥85 mmHg, or taking prescription for hypertension; (3) hypertriglyceridemia: triglyceride (TG) ≥150 mg/dL; (4) low high density lipoprotein cholesterol (HDLC): HDL-C <40 mg/dL for males and <50 mg/dL for females; and (5) elevated blood glucose: fasting glucose ≥100 mg/dL or taking insulin or diabetic pills to lower blood sugar.

### 2.5 Data cleaning

Before conducting the calculations, we implemented a data cleaning step to ensure the accuracy of our consumption estimates. Specifically, any negative values reported for alcohol consumption—considered to be data entry errors, invalid responses, or instances where patients chose not to answer—were excluded from the analysis. This approach was consistently applied across all alcohol consumption variables to maintain uniformity in data processing.

### 2.6 Statistical analysis

We presented baseline characteristics in the form of means (standard deviation) or median [interquartile range (IQR)] for quantitative variables and in the form of frequencies [percentages (%)] for categorical variables. Differences in characteristics were compared by using ANOVA tests or the Kruskal–Wallis *H* test for continuous variables and chi-squared tests for categorical variables.

Multivariable logistic regression models were employed to estimate the association between changes in alcohol consumption and hepatic steatosis, with results reported as odds ratios (ORs) and corresponding 95% confidence intervals (CIs). The analyses included the following adjustments: the crude model adjusted for no covariates. Model 1 adjusted for age and sex. Model 2 adjusted for the same risk factors as Model 1 plus BMI, waist circumference (WC), hypertension (HP), diabetes, hypertension medication, diabetes medication, dyslipidemia medication. Model 3 included all the factors in Model 2, further adjusted for aspartate aminotransferase (AST), alanine aminotransferase (ALT), gamma-glutamyl transferase (GGT), albumin, triglycerides, HDL, HbA1c. Additionally, we further conducted stratified analyses based on demographics and comorbidities, specifically age (<65 years versus ≥65 years), sex (male versus female), BMI (<30 kg/m^2^ versus ≥30 kg/m^2^), and HP (yes versus no). In these analyses, models were controlled for all other covariates except those used for stratification to examine the potential modifiers of effect.

We did not use any imputation method for missing data due to the low rate of missing values. All tests were two-tailed, and *p* < 0.05 was considered significant. Statistical analyses were conducted by using R software (version 4.4.1, The R Foundation for Statistical Computing, Vienna, Austria).

## 3 Results

### 3.1 Baseline characteristics

According to the inclusion and exclusion criteria, a total of 33,427 participants (50.32% male, mean age: 55.13 ± 7.49 years) were included in the baseline alcohol use status analyses. The baseline characteristics of these participants are presented in Table 1. Among the 33,427 participants, 22,370 (66.92%) were classified as mild alcohol consumers, 8,552 (25.58%) as moderate alcohol consumers, and 2,505 (7.5%) as heavy alcohol consumers. Overall, participants who were classified as heavy alcohol consumers were more likely to be younger, female, and had a higher prevalence of previous and current smoking. Additionally, they exhibited higher levels of BMI, systolic blood pressure (SBP), diastolic blood pressure (DBP), WC, as well as elevated platelet counts, glucose, AST, ALT, GGT, triglycerides, and HDL.

**Table 1:** Baseline characteristics of participants for baseline alcohol consumption status analyses.

Variable	Overall	Mild alcohol consumption	Moderate alcohol consumption	Heavy alcohol consumption	*p*-value
Total, *n* (%)	33,427	22,370 (66.92)	8,552 (25.58)	2,505 (7.5)	
Baseline age, years, mean (SD)	55.13 ± 7.49	55.09 ± 7.59	55.28 ± 7.32	54.97 ± 7.21	0.082
Sex, male, *n* (%)	16,820 (50.32)	11,117 (49.70)	4,491 (52.51)	1,212 (48.38)	<0.001
BMI, kg/m^2^, mean (SD)	26.41 ± 4.00	26.32 ± 4.05	26.44 ± 3.82	27.04 ± 4.03	<0.001
Waist circumference, cm, mean (SD)	87.94 ± 12.27	87.52 ± 12.27	88.41 ± 12.10	90.06 ± 12.48	<0.001
Hip circumference, cm, mean (SD)	101.92 ± 7.75	101.80 ± 7.84	101.97 ± 7.43	102.86 ± 7.88	<0.001
Smoking status, *n* (%)					<0.001
Never	19,746 (59.07)	14,611 (65.32)	4,258 (49.79)	877 (35.01)	
Previous	11,546 (34.54)	6,649 (29.72)	3,604 (42.14)	1,293 (51.62)	
Current	2,073 (6.20)	1,077 (4.81)	668 (7.81)	328 (13.09)	
Prefer not to answer	61 (0.18)	32 (0.14)	22 (0.26)	7 (0.28)	
Systolic blood pressure, mmHg, mean (SD)	137.35 ± 18.74	136.49 ± 17.90	138.43 ± 18.20	141.02 ± 18.64	<0.001
Diastolic blood pressure, mmHg, mean (SD)	81.56 ± 10.41	80.94 ± 9.97	82.34 ± 10.08	83.94 ± 10.12	<0.001
Hypertension, *n* (%)	7,475 (22.36)	4,708 (21.05)	2,045 (23.91)	722 (28.82)	<0.001
Diabetes mellitus, *n* (%)	1,964 (5.88)	1,354 (6.05)	446 (5.22)	164 (6.55)	0.007
Antihypertensive medication, *n* (%)	2,825 (8.45)	1,843 (8.24)	727 (8.50)	255 (10.18)	0.004
Glucose-lowering drug, *n* (%)	99 (0.30)	71 (0.32)	23 (0.27)	5 (0.20)	0.510
Lipid-lowering therapy, *n* (%)	3,035 (9.08)	1,933 (8.64)	858 (10.03)	244 (9.74)	<0.001
Platelet, 10^9^/L, mean (SD)	248.60 ± 55.86	248.09 ± 55.05	249.17 ± 53.10	250.79 ± 55.06	0.032
Glucose, mmol/L, mean (SD)	5.00 ± 0.93	4.98 ± 0.84	5.03 ± 0.88	5.09 ± 0.95	<0.001
AST, U/L, mean (SD)	25.83 ± 10.00	25.55 ± 9.80	26.01 ± 8.78	26.96 ± 11.01	<0.001
ALT, U/L, mean (SD)	23.00 ± 13.73	22.67 ± 13.07	23.44 ± 13.48	24.44 ± 14.39	<0.001
GGT, U/L, mean (SD)	34.11 ± 33.50	31.71 ± 27.65	36.91 ± 35.82	45.84 ± 50.86	<0.001
Albumin, g/L, mean (SD)	45.50 ± 2.51	47.22 ± 4.98	47.48 ± 4.99	47.56 ± 4.95	<0.001
Triglycerides, mmol/L, median (IQR)	1.38 (0.98–2.00)	1.46 (1.02–1.95)	1.42 (0.99–1.91)	1.46 (0.99–2.00)	0.014
High-density lipoprotein, mmol/L, mean (SD)	1.50 ± 0.38	1.46 ± 0.34	1.55 ± 0.36	1.64 ± 0.40	<0.001
Hemoglobin A1c, mmol/L, mean (SD)	34.92 ± 5.10	34.97 ± 5.00	34.63 ± 4.69	34.58 ± 4.88	<0.001

Baseline characteristics are represented as mean (standard deviation), median (interquartile range) or *N* (%).

BMI, body mass index; IQR, interquartile range; SD, standard deviation; AST, aspartate aminotransferase; ALT, alanine aminotransferase; GGT, gamma-glutamyl transferase.

In the imaging follow-up session, conducted after a median follow-up period of 9.2 years, a significant proportion of participants exhibited notable changes in their alcohol consumption patterns. Among the 33,427 participants, 25,181 (75.33%) were classified as mild alcohol consumers, 6,449 (19.29%) as moderate alcohol consumers, and 1,797 (5.38%) as heavy alcohol consumers. The baseline characteristics of participants during the imaging visit were similar to those reported in Supplementary Table S4.

### 3.2 Association of baseline alcohol consumption with hepatic steatosis

Supplementary Tables S2, S3 illustrate the association between baseline alcohol consumption and hepatic steatosis. Of the 33,427 participants, 9,131 (27.3%) were diagnosed with hepatic steatosis. After adjusting for confounders, participants with heavy and moderate alcohol consumption displayed significantly higher odds of developing hepatic steatosis compared to those with mild alcohol consumption, with adjusted aORs of 1.41 (95% CI: 1.31–1.52) and 2.60 (95% CI: 2.30–2.93), respectively.

### 3.3 Association between changes in alcohol consumption with hepatic steatosis

**Figure 2:**
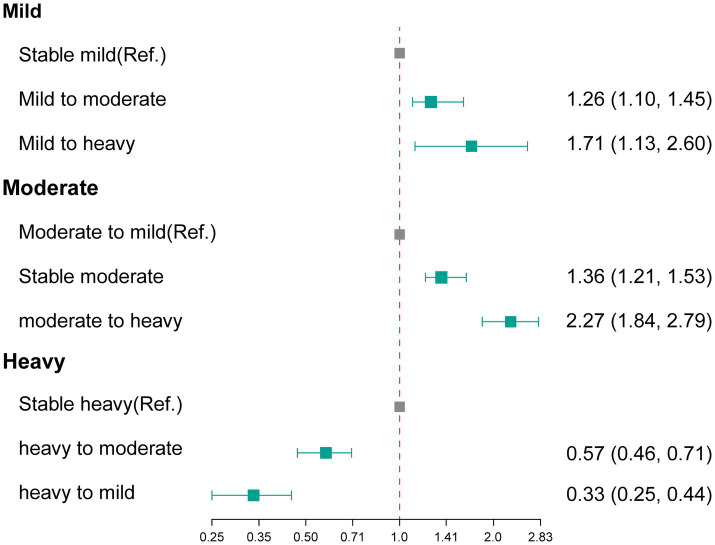
The association between changes in alcohol consumption with hepatic steatosis. Ref., reference group.

Table 2 presents the number and percentage of changes in alcohol consumption status during the imaging follow-up period. Among participants classified as having mild alcohol consumption at baseline, 1,734 participants (7.75%) progressed to moderate or heavy drinking. Among those categorized as moderate drinkers at baseline, 4,081participants (47.72%) transitioned to mild drinking, while 620 participants (7.25%) advanced to heavy drinking. Additionally, among participants identified as heavy drinkers at baseline, 464participants (18.52%) downgraded to mild drinking, and 1,016 participants (40.56%) shifted to moderate drinking. Figure 2 and Supplementary Table S4 shows the association between changes in alcohol consumption with hepatic steatosis. In comparison with stable mild participants, mild participants who progressed to moderate status or heavy status showed significantly elevated odds of developing hepatic steatosis, the aORs were 1.26 (95% CI: 1.10–1.44) and 1.70 (95% CI: 1.12–2.57), respectively. In addition, in comparison with participants who transitioned to mild status, moderate participants who stable moderate or progressed to heavy status showed significantly elevated odds of developing hepatic steatosis, the aORs were 1.36 (95% CI: 1.21–1.53) and 2.27 (95% CI: 1.84–2.79), respectively. In contrast, significantly decreased odds of developing hepatic steatosis were observed in the heavy participants who transitioned to moderate/mild status when compared with stable heavy participants (heavy to moderate, aOR 0.58, 95% CI: 0.47–0.72; heavy to mild, aOR 0.34, 95% CI: 0.25–0.45).

**Table 2:** Number and percentage of the changes in alcohol consumption status.

Baseline status	The imaging visit status	Changes in alcohol consumption status	*n* (%)
Mild	Mild	Stable mild	20,636 (92.25)
Mild	Moderate	Mild to moderate	1,582 (7.07)
Mild	Heavy	Mild to heavy	152 (0.68)
Moderate	Mild	Moderate to mild	4,081 (47.72)
Moderate	Moderate	Stable moderate	3,851 (45.03)
Moderate	Heavy	Moderate to heavy	620 (7.25)
Heavy	Mild	Heavy to mild	464 (18.52)
Heavy	Moderate	Heavy to moderate	1,016 (40.56)
Heavy	Heavy	Stable heavy	1,025 (40.92)

The median follow-up time between baseline and the imaging visit was 9.2 years.

### 3.4 Stratified analysis

**Figure 3:**
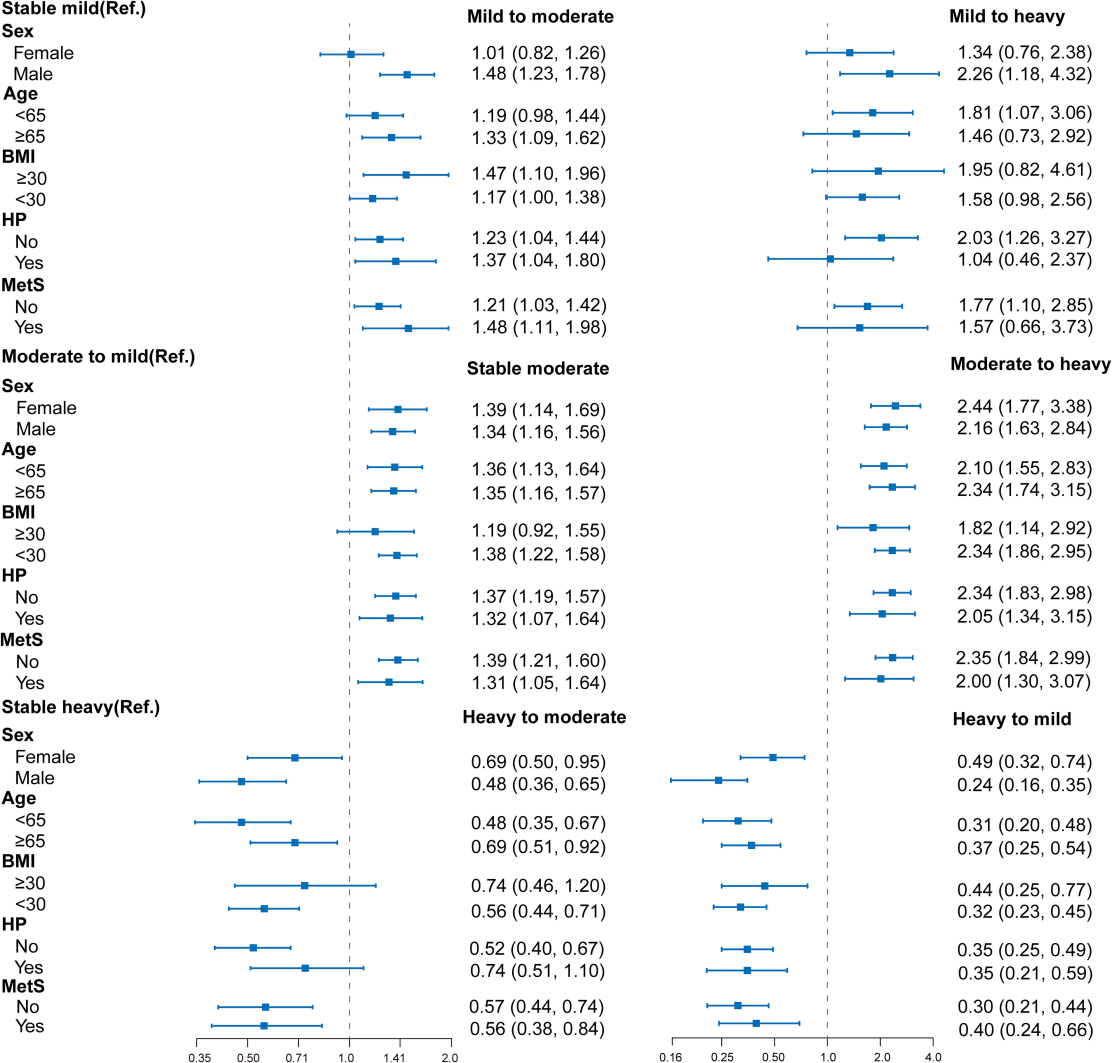
Stratified analysis for the association between changes in alcohol consumption and hepatic steatosis. Multivariable logistic regression models were adjusted for all the factors (age, sex, hypertension, BMI, waist circumference, diabetes mellitus, antihypertensive medication, glucose-lowering drug, and lipid-lowering therapy, AST, ALT, GGT, albumin, triglycerides, HDL, HbA1c and duration of alcohol consumption.) except the stratification factor itself. BMI, body mass index; HP, hypertension; OR, hazard ratio; Ref., reference group.

Figure 3 and Supplementary Table S5 presents a stratified analysis for the association between changes in alcohol consumption and hepatic steatosis among alcohol consumers, highlighting significant effects of sex, age, BMI, and hypertension status on this relationship. Males exhibited a notably higher risk of transitioning from stable mild to heavy drinking, with an OR of 2.26, while females demonstrated a lower risk in the transition from heavy to mild drinking (OR of 0.24). In terms of age, individuals under 65 showed an increased risk of moving from mild to heavy drinking (OR of 1.81), whereas those aged 65 and older had a relatively lower risk. Furthermore, individuals with a BMI of 30 or higher faced significantly heightened risk in the transition from mild to heavy alcohol consumption (OR of 1.95), and hypertensive patients also displayed an increased risk across various transitions, particularly from mild to heavy drinking (OR of 2.34). These findings suggest that males, younger individuals, those with higher BMI, and patients with hypertension are more susceptible to severe hepatic steatosis as alcohol consumption increases.

## 4 Discussion

Our comprehensive analysis of a large UK-based prospective study showed that participants who were heavy drinkers at baseline but reduced their alcohol consumption during the imaging follow-up period experienced significantly lower odds of developing hepatic steatosis. In contrast, participants who were mild drinkers at baseline but increased their alcohol consumption during the imaging follow-up period demonstrated significantly higher odds of developing hepatic steatosis. This association remained consistent across a series of sensitivity analyses. This study is the first to investigate the impact of changes in alcohol consumption over time on hepatic steatosis in a large population cohort. Our findings suggest that assessing alcohol consumption and implementing early interventions may help reduce the risk of developing hepatic steatosis, particularly among participants with heavy or moderate alcohol consumption.

The relationship between alcohol consumption and hepatic steatosis in population-based studies has been inconsistent, as highlighted by previous research (31). For instance, a cross-sectional study involving 4,009 participants from northeastern Germany demonstrated a significant increase in the risk of hepatic steatosis associated with higher average daily alcohol intake in both men and women, particularly among those who were overweight or obese. Notably, heavy alcohol consumption exacerbated this risk in individuals with excess body weight (32). In contrast, a longitudinal study of 5,297 Japanese participants (3,773 men and 1,524 women) suggested that mild to moderate alcohol consumption—and even slightly higher levels in men—may confer long-term protection against fatty liver for the majority of individuals (10). The study by Hagström et al. (33) also showed that low to moderate lifetime alcohol consumption is associated with less advanced stages of fibrosis in non-alcoholic fatty liver disease. Our findings reveal a clear dose–response relationship between alcohol intake and hepatic steatosis among drinkers, indicating that the risk of hepatic steatosis escalates as alcohol consumption increases. Conversely, a reduction in alcohol intake was associated with a decreased likelihood of developing hepatic steatosis. These results are consistent with the findings reported by Bedogni et al. (34). Collectively, our study strengthens the evidence that elevated alcohol consumption is a potential risk factor for hepatic steatosis.

The complex mechanisms underlying the bidirectional relationship between alcohol consumption and hepatic steatosis remain incompletely understood. The liver serves as the central hub for systemic lipid metabolism, processing free fatty acids (FAs) derived from multiple sources including glycolysis, autophagy, and adipose tissue lipolysis. These FAs are subsequently metabolized through β-oxidation for energy production, incorporated into cellular membranes, or esterified into triglycerides within hepatocytes. The synthesized triglycerides are then either packaged into very low-density lipoproteins (VLDLs) for systemic distribution or serve as precursors for primary bile acid synthesis, which facilitates dietary lipid emulsification and absorption. This intricate metabolic network is tightly regulated by hormonal signals, nuclear receptors, and intracellular signaling pathways, maintaining hepatic lipid homeostasis under physiological conditions. However, disruption of these regulatory mechanisms can lead to pathological lipid accumulation in hepatocytes, resulting in fatty liver disease. Additionally, changes in gut microbiota [such as reduced levels of short-chain fatty acids (SCFAs) and decreased bacterial diversity] play a key role in the development and progression of fatty liver disease (35). Alcohol exerts multifaceted effects on hepatic lipid flux through both direct and indirect mechanisms. During alcohol consumption, blood alcohol concentrations can reach millimolar levels (9), triggering a cascade of metabolic alterations. Ethanol metabolism generates reduced NADH, significantly increasing the hepatic NADH:NAD^+^ ratio, which simultaneously inhibits fatty acid β-oxidation while promoting fatty acid esterification (36). Furthermore, the primary ethanol metabolite acetaldehyde disrupts lipid homeostasis by upregulating key lipogenic genes, including Srebf1, Fasn, and Acc1, through enhanced nuclear translocation and subsequent transcriptional activation of their target genes (37, 38). These alcohol-induced metabolic perturbations collectively promote intrahepatocytic triglyceride accumulation, ultimately driving the pathogenesis of hepatic steatosis. These mechanistic insights are consistent with our study findings, which demonstrate a dose-dependent relationship between alcohol consumption and the risk of hepatic steatosis development.

Abstinence is the most critical intervention for treating hepatic steatosis. However, the improvement in hepatic steatosis after the cessation of alcohol consumption is inconsistent; some patients may still progress to cirrhosis or experience liver decompensation even while remaining abstinent (37, 39). Research conducted by Schonfeld et al. (40) identified the hepatic demethylases lysine demethylase (KDM) 5B and KDM5C as important epigenetic regulators of the liver’s response to alcohol. These enzymes hinder the resolution of liver fibrosis after alcohol cessation, partly by suppressing liver X receptor (LXR) activity. Additionally, Kang et al. (41) performed a mouse model study that demonstrated a gradual decrease in the expression of lipogenic genes following ethanol withdrawal. They also found that hepatic NAD^+^ levels were rapidly restored after stopping ethanol intake, reaching levels comparable to those in the no-ethanol control group by the third week of the withdrawal period. This evidence suggests that liver steatosis significantly diminishes after abstaining from alcohol. Furthermore, abstaining from alcohol can effectively restore lipid metabolism in the liver of mice, reversing liver damage and inflammation through enhanced metabolic reprogramming (42, 43). Data from our study indicate that among drinkers, a reduction in alcohol consumption significantly lowers the likelihood of developing hepatic steatosis. This highlights that abstaining from alcohol or reducing intake is an effective strategy for managing hepatic steatosis.

To the best of our knowledge, this study is the first to investigate the relationship between changes in alcohol consumption and hepatic steatosis. The strengths of our research include the utilization of a large longitudinal cohort dataset, which features high-quality data collected through standardized methods and multiple measurements, enabling us to control for a comprehensive range of confounding variables. However, there are several limitations to note. Firstly, the cross-sectional design of the study restricts our ability to infer causality regarding the effects of changes in alcohol consumption on hepatic steatosis, making it difficult to determine the direction of the relationship between these two factors. Secondly, we did not conduct liver biopsies, which are considered the gold standard for diagnosing fatty liver disease. Nevertheless, our study employed MRI to evaluate hepatic fat content, which yields reliable results. Thirdly, although the adapted measurements have been validated, alcohol consumption was assessed using self-reported data, which may introduce recall bias and misclassification. Fourthly, the UK Biobank participants tend to be ethnically and racially homogeneous, which limits the generalizability of our findings; further studies are needed to validate our results in more diverse populations. Fifthly, we did not assess the relationship between alcohol consumption and factors such as meal composition, total caloric intake, types of beverages (e.g., beer, wine), or educational level. Lastly, there may be unmeasured confounders, particularly those that are not identifiable through routine health screenings, such as stress or other mental health factors.

## 5 Conclusion

Our findings demonstrate a significant association between changes in alcohol consumption and hepatic steatosis, highlighting that early interventions to modify drinking behaviors may represent an effective strategy for preventing hepatic steatosis.

## Data Availability

The raw data supporting the conclusions of this article will be made available by the authors, without undue reservation.

## Footnotes

^1^ http://www.ukbiobank.ac.uk

## Glossary

ABV: Alcohol by volume
ALD: Alcohol-related liver disease
ALT: Alanine aminotransferase
AST: Aspartate aminotransferase
BMI: Body mass index
CAP: Controlled attenuation parameter
CI: Confidence interval
DBP: Diastolic blood pressure
FLD: Fatty liver disease
GGT: Gamma-glutamyl transferase
HbA1c: Glycated hemoglobin
HDL: High-density lipoprotein
HP: Hypertension
LDL: Low-density lipoprotein
OR: Hazard ratio
IQR: Interquartile range
MASLD: Metabolic dysfunction-associated steatotic liver disease
MRI: Magnetic resonance imaging
PDFF: Proton density fat fraction
SBP: Systolic blood pressure
SD: Standard deviation
UK: Biobank United Kingdom Biobank
WC: Waist circumference
