# Supplementary material for: The relationship between changes in alcohol consumption and hepatic steatosis among alcohol consumers: a large-scale population-based Biobank study
Source: Front Nutr. 2025 Oct 16;12:1647225. doi: 10.3389/fnut.2025.1647225 (PMC12571616; doi:10.3389/fnut.2025.1647225)
Supplement: Supplementary file 1 [file Table_1.docx]

**Supplementary Online Content**

**Table S1. Baseline characteristics of participants for changes in alcohol consumption status analyses**

**Table S2. Univariable analysis of baseline characteristics with hepatic steatosis**

**Table S3. Multivariable analysis of baseline alcohol consumption with hepatic steatosis**

**Table S4. Multivariable analysis for changes in alcohol consumption status with hepatic steatosis**

**Table S5. Stratified analysis for the association between changes in alcohol consumption and hepatic steatosis among alcohol consumers**

| **Table S1. Baseline characteristics of participants for changes in alcohol consumption status analyses** | | | | | |
| --- | --- | --- | --- | --- | --- |
| **Variable** | **Overall** | **Mild alcohol consumption** | **Moderate alcohol consumption** | **Heavy alcohol consumption** | ***P* value** |
| **Total, n(%)** | 33,427 | 25,181（75.33） | 6,449（19.29） | 1,797（5.38） |  |
| **Baseline age, years, mean (SD)** | 64.58 ± 7.63 | 64.74 ± 7.69 | 64.25 ± 7.44 | 63.37 ± 7.29 | <0.001 |
| **Sex, male, n(%)** | 16,820 (50.32) | 12,663 (50.29) | 3,320 (51.48) | 837 (46.58) | 0.001 |
| **Smoking status, n (%)** |  |  |  |  | <0.001 |
| Never | 20,325 (60.80) | 16,457 (65.35) | 3,139 (48.67) | 729 (40.57) |  |
| Previous | 11,864 (35.49) | 7,968 (31.64) | 2,977 (46.16) | 919 (51.14) |  |
| Current | 1,138 (3.40) | 681 (2.70) | 313 (4.85) | 144 (8.01) |  |
| Prefer not to answer | 100 (0.30) | 75 (0.30) | 20 (0.31) | 5 (0.28) |  |
| **Systolic blood pressure, mmHg, mean (SD)** | 141.20 ± 17.66 | 140.78 ± 17.63 | 142.22 ± 17.55 | 143.40 ± 18.14 | <0.001 |
| **Diastolic blood pressure, mmHg, mean (SD)** | 78.68 ± 9.46 | 78.40 ± 9.42 | 79.38 ± 9.41 | 80.21 ± 9.82 | <0.001 |
| **Waist circumference, cm, mean (SD)** | 88.11 ± 12.19 | 87.75 ± 12.17 | 88.92 ± 12.06 | 90.28 ± 12.64 | <0.001 |
| **BMI, kg/m^2^, mean (SD)** | 26.30 ± 4.07 | 26.20 ± 4.10 | 26.46 ± 3.88 | 27.06 ± 4.27 | <0.001 |
| **Hip circumference, cm, mean (SD)** | 100.41 ± 8.20 | 100.26 ± 8.27 | 100.63 ± 7.76 | 101.68 ± 8.63 | <0.001 |
| **Duration of alcohol consumption, year, mean (SD)** | 8.97 ± 1.71 | 8.96 ± 1.72 | 9.01 ± 1.71 | 9.00 ± 1.69 | 0.028 |
| **PDFF** |  |  |  |  | <0.001 |
| ＜5% | 24,296(62.42) | 18,846 (74.84) | 4,444 (68.91) | 1,006 (55.98) |  |
| ≥5% | 9,131(37.58) | 6,335 (25.16) | 2,005 (31.09) | 791 (44.02) |  |
| Baseline characteristics are represented as mean (standard deviation) or N (%). | | | | | |

| **Table S2. Univariable analysis of baseline characteristics with hepatic steatosis** | | | | | |
| --- | --- | --- | --- | --- | --- |
| **Variable** | **PDFF ＜5%(n = 24,296)** | **PDFF ≥5%(n =9,131)** | **OR** | **95%CI** | ***p* value** |
| **Sex** |  |  |  |  |  |
| Female | 13,313 (54.80) | 3,294 (36.07) | 1 |  |  |
| Male | 10,983 (45.20) | 5,837 (63.93) | 2.15 | 2.04-2.26 | <0.0001 |
| **Age at imaging visit** | 64.49 ± 7.71 | 64.81 ± 7.41 | 1.01 | 1.00-1.01 | 0.0006 |
| **Smoking status** |  |  |  |  |  |
| Never | 15,173 (62.45) | 5,152 (56.42) | 1 |  |  |
| Previous | 8,266 (34.02) | 3,598 (39.40) | 1.28 | 1.22-1.35 | <0.0001 |
| Current | 786 (3.24) | 352 (3.85) | 1.32 | 1.16-1.50 | <0.0001 |
| Prefer not to answer | 71 (0.29) | 29 (0.32) | 1.20 | 0.78-1.85 | 0.4031 |
| **Hypertension** |  |  |  |  |  |
| No | 19,791 (81.46) | 6,161 (67.47) | 1 |  |  |
| Yes | 4,505 (18.54) | 2,970 (32.53) | 2.12 | 2.01-2.24 | <0.0001 |
| **Diabetes mellitus** |  |  |  |  |  |
| No | 23,567 (97.00) | 8,108 (88.80) | 1 |  |  |
| Yes | 729 (3.00) | 1,023 (11.20) | 4.08 | 3.70-4.50 | <0.0001 |
| **Antihypertensive medication** |  |  |  |  |  |
| No | 22,607 (93.05) | 7,995 (87.56) | 1 |  |  |
| Yes | 1,689 (6.95) | 1,136 (12.44) | 1.90 | 1.76-2.06 | <0.0001 |
| **Glucose-lowering drug** |  |  |  |  |  |
| No | 24,239 (99.77) | 9,089 (99.54) | 1 |  |  |
| Yes | 57 (0.23) | 42 (0.46) | 1.97 | 1.32-2.93 | 0.0009 |
| **Lipid-lowering therapy** |  |  |  |  |  |
| No | 22,547 (92.80) | 7,845 (85.92) | 1 |  |  |
| Yes | 1,749 (7.20) | 1,286 (14.08) | 2.11 | 1.96-2.28 | <0.0001 |
| **Alcohol consumption status** |  |  |  |  |  |
| Mild | 18,846 (77.57) | 6,335 (69.38) | 1 |  |  |
| Moderate | 4,444 (18.29) | 2,005 (21.96) | 1.34 | 1.26-1.43 | <0.0001 |
| Heavy | 1,006 (4.14) | 791 (8.66) | 2.34 | 2.12-2.58 | <0.0001 |
| **BMI** | 25.50 ± 3.58 | 28.83 ± 4.06 | 1.25 | 1.24, 1.26 | <0.0001 |
| **Waist circumference at imaging visit** | 84.72 ± 10.79 | 97.14 ± 11.06 | 1.11 | 1.11-1.11 | <0.0001 |
| **AST** | 24.98 ± 9.13 | 27.89 ± 10.66 | 1.04 | 1.04-1.04 | <0.0001 |
| **ALT** | 20.69 ± 11.05 | 29.15 ± 16.41 | 1.06 | 1.06-1.06 | <0.0001 |
| **GGT** | 30.38 ± 28.22 | 44.00 ± 39.87 | 1.01 | 1.01-1.02 | <0.0001 |
| **Albumin** | 45.35 ± 2.32 | 45.63 ± 2.34 | 1.05 | 1.04-1.06 | <0.0001 |
| **High-density lipoprotein** | 1.55 ± 0.35 | 1.36 ± 0.31 | 0.16 | 0.15-0.17 | <0.0001 |
| **Triglycerides** | 1.47 ± 0.78 | 2.07 ± 1.10 | 2.03 | 1.97-2.09 | <0.0001 |
| **Glucose** | 4.95 ± 0.78 | 5.12 ± 1.03 | 1.23 | 1.20-1.27 | <0.0001 |
| Baseline characteristics are represented as mean (standard deviation) or N (%). | | | | | |
| BMI,body mass index; IQR, interquartile range; SD, standard deviation; AST, aspartate aminotransferase; ALT, alanine aminotransferase; GGT, gamma-glutamyl transferase. | | | | | |

| **Table S3. Multivariable analysis of baseline alcohol consumption with hepatic steatosis** | | |
| --- | --- | --- |
|  | OR(95%CI)^a^ | *P*^a^ |
| mild | 1(reference) |  |
| moderate | 1.41 (1.31, 1.52) | <0.0001 |
| heavy | 2.60 (2.30, 2.93) | <0.0001 |
| ^a^OR and *P* were adjusted for age, sex, smoking status, body mass index, hypertension, diabetes mellitus, waist circumference, AST, ALT, GGT, Albumin, high-density lipoprotein cholesterol, triglycerides, glucose, antihypertensive medication, glucose-lowering drug, and lipid-lowering therapy. | | |
| AST, aspartate aminotransferase; ALT, alanine aminotransferase; GGT, gamma-glutamyl transferase. | | |

| **Table S4. Multivariable analysis for changes in alcohol consumption status with hepatic steatosis** | | | | | | | | | |
| --- | --- | --- | --- | --- | --- | --- | --- | --- | --- |
| **Baseline status** | **The imaging visit status** | **Crude model** | | **Model 1** | | **Model 2** | | **Model 3** | |
|  |  | **OR[95% CI]** | ***P* value** | **OR[95% CI]** | ***P* value** | **OR[95% CI]** | ***P* value** | **OR[95% CI]** | ***P* value** |
| Mild | Stable mild | 1[ref. category] |  | 1[ref. category] |  | 1[ref. category] |  | 1[ref. category] |  |
|  | Mild to moderate | 1.17 (1.04, 1.31) | 0.008 | 1.22 (1.09, 1.37) | 0.001 | 1.17 (1.02-1.33) | 0.024 | 1.26 (1.10, 1.45) | 0.001 |
|  | Mild to heavy | 1.44 (1.02, 2.02) | 0.037 | 1.66 (1.17, 2.34) | 0.004 | 1.52 (1.02, 2.27) | 0.042 | 1.71 (1.13, 2.60) | 0.012 |
| Moderate | Moderate to mild | 1[ref. category] |  | 1[ref. category] |  | 1[ref. category] |  | 1[ref. category] |  |
|  | Stable moderate | 1.25 (1.13, 1.38) | <0.0001 | 1.23 (1.11, 1.36) | <0.0001 | 1.29 (1.15, 1.44) | <0.0001 | 1.36 (1.21, 1.53) | <0.0001 |
|  | moderate to heavy | 2.01 (1.69, 2.40) | <0.0001 | 2.13 (1.78, 2.55) | <0.0001 | 2.13 (1.74, 2.61) | <0.0001 | 2.27 (1.84, 2.79) | <0.0001 |
| Heavy | Stable heavy | 1[ref. category] |  | 1[ref. category] |  | 1[ref. category] |  | 1[ref. category] |  |
|  | heavy to moderate | 0.65 (0.55, 0.78) | <0.0001 | 0.63 (0.52, 0.75) | <0.0001 | 0.62 (0.51, 0.77) | <0.0001 | 0.57 (0.46, 0.71) | <0.0001 |
|  | heavy to mild | 0.42 (0.33, 0.54) | <0.0001 | 0.41 (0.32, 0.52) | <0.0001 | 0.37 (0.28, 0.49) | <0.0001 | 0.33 (0.25, 0.44) | <0.0001 |
| Crude model adjusted for none. | | | | | | | | | |
| Model 1 adjusted for age, sex. | | | | | | | | | |
| Model 2 adjusted for the same risk factors as Model 1 and body mass index, waist circumference, hypertension, diabetes mellitus, antihypertensive medication, glucose-lowering drug, and lipid-lowering therapy. | | | | | | | | | |
| Model 3 adjusted for the same risk factors as Model 2 and AST, ALT, GGT, Albumin, Triglycerides, HDL, HbA1c and duration of alcohol consumption. | | | | | | | | | |

| **Table S5. Stratified analysis for the association between changes in alcohol consumption and hepatic steatosis among alcohol consumers** | | | | | | | | | |
| --- | --- | --- | --- | --- | --- | --- | --- | --- | --- |
| **Variable** | **mild** | | | **Moderate** | | | **Heavy** | | |
|  | **Stable mild** | **Mild to moderate** | **Mild to heavy** | **Moderate to mild** | **Stable moderate** | **moderate to heavy** | **Stable heavy** | **heavy to moderate** | **heavy to mild** |
| **Sex** |  |  |  |  |  |  |  |  |  |
| Female | Ref. | 1.02 (0.82, 1.27) | 1.37 (0.77, 2.42) | Ref. | 1.38 (1.14, 1.68) | 2.43 (1.76, 3.36) | Ref. | 0.68 (0.49, 0.94) | 0.48 (0.32, 0.73) |
| Male | Ref. | 1.49 (1.24, 1.78) | 2.25 (1.18, 4.29) | Ref. | 1.35 (1.16, 1.56) | 2.16 (1.64, 2.85) | Ref. | 0.48 (0.35, 0.64) | 0.23 (0.16, 0.35) |
| **Age** |  |  |  |  |  |  |  |  |  |
| <65 | Ref. | 1.19 (0.98, 1.44) | 1.82 (1.08, 3.08) | Ref. | 1.37 (1.14, 1.64) | 2.10 (1.55, 2.83) | Ref. | 0.48 (0.35, 0.67) | 0.31 (0.20, 0.47) |
| >=65 | Ref. | 1.34 (1.10, 1.63) | 1.44 (0.72, 2.89) | Ref. | 1.35 (1.16, 1.57) | 2.34 (1.75, 3.15) | Ref. | 0.67 (0.50, 0.90) | 0.36 (0.24, 0.53) |
| **BMI** |  |  |  |  |  |  |  |  |  |
| >=30 | Ref. | 1.48 (1.10, 1.97) | 1.98 (0.84, 4.70) | Ref. | 1.19 (0.92, 1.54) | 1.82 (1.14, 2.91) | Ref. | 0.73 (0.45, 1.19) | 0.43 (0.24, 0.76) |
| <30 | Ref. | 1.18 (1.01, 1.39) | 1.59 (0.98, 2.58) | Ref. | 1.38 (1.21, 1.58) | 2.34 (1.86, 2.95) | Ref. | 0.55 (0.43, 0.70) | 0.31 (0.22, 0.44) |
| **HP** |  |  |  |  |  |  |  |  |  |
| No | Ref. | 1.23 (1.05, 1.45) | 2.04 (1.26, 3.29) | Ref. | 1.37 (1.19, 1.57) | 2.33 (1.83, 2.97) | Ref. | 0.51 (0.39, 0.67) | 0.34 (0.24, 0.48) |
| Yes | Ref. | 1.38 (1.05, 1.81) | 1.06 (0.46, 2.41) | Ref. | 1.34 (1.08, 1.66) | 2.06 (1.34, 3.17) | Ref. | 0.73 (0.50, 1.08) | 0.35 (0.20, 0.58) |
| **MetS** |  |  |  |  |  |  |  |  |  |
| No | Ref. | 1.21 (1.03, 1.42) | 1.77 (1.10, 2.85) | Ref. | 1.39 (1.21, 1.60) | 2.35 (1.84, 2.99) | Ref. | 0.57 (0.44, 0.74) | 0.30 (0.21, 0.44) |
| Yes | Ref. | 1.48 (1.11, 1.98) | 1.57 (0.66, 3.73) | Ref. | 1.31 (1.05, 1.64) | 2.00 (1.30, 3.07) | Ref. | 0.56 (0.38, 0.84) | 0.40 (0.24, 0.66) |
| The association between changes in alcohol consumption and hepatic steatosis among alcohol consumers are represented as OR[95% CI] . Each stratification was adjusted for all the factors (age, sex, hypertension, BMI, waist circumference,diabetes mellitus, antihypertensive medication, glucose-lowering drug, and lipid-lowering therapy, AST, ALT, GGT, Albumin, triglycerides, HDL, HbA1c and duration of alcohol consumption.) except the stratification factor itself. | | | | | | | | | |
